# Maternal Group B Streptococcal (GBS) Genital Tract Colonization at Term in Women who Have Asymptomatic GBS Bacteriuria

**DOI:** 10.1080/10647440300025522

**Published:** 2003

**Authors:** David S. McKenna, Scott Matson, Ike Northern

**Affiliations:** 1 Department of Obstetrics and Gynecology Wright State University 74th Medical Group/SGOG 4881 Sugar Maple Dr., Wright-Patterson AFB Dayton OH 45433 USA; 2 Compunet Clinical Laboratories Dayton OH USA

## Abstract

*Objective:* To determine the rate of positive group B streptococcus (GBS) cultures at 35–37 weeks gestation in women who have first trimester asymptomatic GBS bacteriuria.

*Methods:* Pregnant women with asymptomatic first trimester GBS bacteriuria had genital cultures for GBS performed at 35–37 weeks gestational age. Serotyping was performed by the standard Lancefield capillary precipitin method.

*Results:*  Fifty-three women with positive urine cultures had genital cultures performed at 35–37 weeks. Sixteen of the 53 (30.2%; 95% confidence interval: 18.4–44.3%) third trimester vaginal cultures were positive for GBS. Five of eight (63%) of the women with typable urine serotypes had the same typable serotype in the third trimester genital culture.

*Conclusion:* Genital tract cultures at 35–37 weeks for GBS correlate poorly with first trimester asymptomatic GBS bacteriuria. Recommendations for GBS prophylaxis in labor in women who have first trimester asymptomatic GBS bacteriuria should be investigated further and reconsidered.


*Infect Dis Obstet Gynecol 2003;11:203–207*

### Maternal group B streptococcal (GBS) genital tract colonization at term in women who have asymptomatic GBS bacteriuria

**David S. McKenna[1], Scott Matson[1] and Ike Northern[2]**

[1]*Department of Obstetrics and Gynecology, Wright State University, Dayton, OH*
[2]*Compunet Clinical Laboratories, Dayton, OH*

***Objective:*** To determine the rate of positive group B streptococcus (GBS) cultures at 35–37 weeks gestation in women who have first trimester asymptomatic GBS bacteriuria.
***Methods:*** Pregnant women with asymptomatic first trimester GBS bacteriuria had genital cultures for GBS performed at 35–37 weeks gestational age. Serotyping was performed by the standard Lancefield capillary precipitin method.
***Results:*** Fifty-three women with positive urine cultures had genital cultures performed at 35–37 weeks. Sixteen of the 53 (30.2%; 95% confidence interval: 18.4–44.3%) third trimester vaginal cultures were positive for GBS. Five of eight (63%) of the women with typable urine serotypes had the same typable serotype in the third trimester genital culture.
***Conclusion:*** Genital tract cultures at 35–37 weeks for GBS correlate poorly with first trimester asymptomatic GBS bacteriuria. Recommendations for GBS prophylaxis in labor in women who have first trimester asymptomatic GBS bacteriuria should be investigated further and reconsidered.

Key words: PREGNANCY; INFECTIONS; PROPHYLAXIS; ANTIBIOTICS; SEROTYPE

Perinatal group B streptococcus (GBS) infection is a major cause of neonatal morbidity and mortality[1]. GBS colonizes the maternal lower genital and gastrointestinal tracts in 20–40% of pregnancies and causes asymptomatic bacteriuria in about 1%[1]. Asymptomatic GBS bacteriuria has been proposed as a marker of heavy genital tract colonization and as a risk factor for adverse perinatal outcome and neonatal disease[1–3]. Edwards and co-workers recently reported first trimester GBS bacteriuria has only a 61% positive predictive value for a positive GBS genital culture at the time of labor[4]. The study by Edwards and co-workers demonstrated that first trimester bacteriuria does not equate to a positive genital culture at the time of labor and raised doubts about the need to give antibiotic prophylaxis based solely on first trimester GBS bacteriuria.

The current perinatal recommendations by the Centers for Disease Control (CDC) call for

Disclaimer: The opinions and conclusions in this paper are those of the authors and are not intended to represent the official position of the Department of Defense, United States Air Force, or any other government agency.

Correspondence to: David S. McKenna, MD, Lt. Col. U.S. Air Force Medical Corp., Wright State University, Department of Obstetrics and Gynecology, 74th Medical Group/SGOG, 4881 Sugar Maple Dr., Wright-Patterson AFB, OH 45433, USA. E-mail: david.mckenna@wpafb.af.mil

© 2003 The Parthenon Publishing Group 

antibiotic prophylaxis for women with asymptomatic first trimester bacteriuria, because this is 'a marker for heavy genital tract colonization', and screening all other women at 35–37 weeks for vaginal and rectal colonization[3]. The rate of GBS genital tract colonization at 35–37 weeks gestation in women who have had first trimester GBS bacteriuria has not been reported and the CDC does not recommend screening these women for GBS at term. The purpose of this study was to determine the rate of GBS genital tract colonization at 35–37 weeks gestation in women who have had a first trimester GBS bacteriuria. The correlation of the specific GBS serotypes in each positive maternal first trimester urine/term genital culture pair was also assessed.

### METHODS

The hospital institutional review board approved the study protocol. The study participants consisted of pregnant women greater than the age of 15 years, receiving prenatal care from the obstetrics and gynecology residents and staff, at the institution's outpatient clinic. All prenatal patients were screened for asymptomatic bacturia during their first prenatal visit, with a standard culture from a self-collected clean catch midstream urine specimen. Asymptomatic bacteriuria was diagnosed when there were at least $10^4$ CFU/ml of a single organism and identification and antibiotic sensitivity of the organism was performed[5]. Positive urine cultures were treated with an appropriate antibiotic and a negative test of cure was confirmed at the next scheduled prenatal visit that occurred at least 1 week after completion of antibiotic therapy. All women with positive GBS cultures had a negative test of cure. If the organism was identified as GBS, then prophylaxis was administered in labor[6]. During the period of the study, genital cultures for GBS were not routinely performed at term and GBS prophylaxis was performed by a risk factor-based approach[6]. Per the clinic's protocol, cervical cultures for gonorrhea/chlamydia were routinely obtained from all pregnant women at 35–37 weeks. Women who had a positive GBS urine culture in the first trimester were eligible to participate in the study. After verbal consent at 35–37 weeks gestational age, specimens for GBS were collected from study participants at the same time as the cervical cultures were obtained.

The genital cultures for GBS were collected prior to performing the speculum exam for the cervical cultures. A standard cotton aerobic bacterial culture swab was gently inserted into the lower third of the vagina and then in a single motion, the perineum was swabbed and the external anal sphincter was swabbed. The swab was then placed in Stuart's transport medium and sent to the laboratory at room temperature. Specimens were maintained at room temperature and all were processed within 4 hours of collection. The swabs were inoculated into 5 ml of Todd Hewitt broth containing 0.1 g/l of colistin and 0.015 g/l of nalidixic acid. The broths were then placed into a 35°C, non-$CO_2$ incubator for 18–24 hours. Broths were next inoculated onto a blood agar plate (trypticase soy agar with 5% sheep blood) and the plates were incubated for 24 hours at 35°C in a 5% $CO_2$ incubator. The plates were then examined for colonies consistent with GBS (milky white, translucent colonies, with or without hemolysis). Serologic identification was then performed on the suspect GBS colonies using a standard commercially available latex agglutination kit (Abbott Diagnostics, Abbott Park IL, USA). If agglutination was absent or if no suspect colonies were present, the blood agar plates were incubated for an additional 24 hours at 35°C in a 5% $CO_2$ incubator. The plates were then re-examined for GBS colonies and if present, serologically identified by latex agglutination. If no colonies were present, the culture was reported negative for GBS. GBS serotyping was performed on the positive urine and genital cultures by the Lancefield capillary precipitin method utilizing antisera to polysaccharide antigens Ia, Ib, II, III, IV and V and protein antigen c prepared at the CDC[7]. The antisera is cross absorbed to ensure that it only reacts with one specific type. Cultures that did not react to the antisera were categorized as non-typable (NT).

The study participants' physicians were blinded to the results of the third trimester genital culture. Outcomes consisted of the fraction of women with positive first trimester GBS urine cultures and positive third trimester genital cultures and the percent of identical serotypes in each positive culture pair. The Fisher Exact Test, with $p < 0.05$ considered significant, was utilized to compare categorical variables. Statistical analysis was performed using GraphPad Prism version 3.0 (GraphPad Software, San Diego CA, USA).

### RESULTS

The study was conducted from January 2001 to March 2002. There were 2318 pregnant women cared for in the clinic study population during this period. Sixty-five first trimester urine cultures were positive for GBS (2.8%). Twelve of the 65 women (18%) with positive urine cultures were no longer being cared for in our clinic by the third trimester and genital cultures for GBS were unable to be obtained from them. The remaining 53 culture pairs were analyzed. The mean age (± standard deviation) was 23.4 ± 5 years. Forty percent of the women were nulliparous. There were 34 African Americans (64%) and 19 (36%) were Caucasian. The mean gestational age at delivery was 39.0 ± 1.6 weeks. There were six women (11.3%) with preterm delivery, four at 36 weeks and one each at 35 and 34 weeks gestation. There were no cases of maternal or neonatal GBS disease.

Sixteen of the 53 third trimester vaginal cultures (30.2%; 95% confidence interval: 18.4–44.3%) were positive for GBS. The serotypes of the positive urine culture and genital cultures are listed in Table 1. Serotypes III (32.1%) and Ia (22.6%) were the most common in the urine cultures. A greater number of urine cultures than vaginal cultures were NT – 16/53 (30.2%) versus 1/16 (6.3%) – but this did not reach statistical significance ($p \leq 0.09$). Table 2 lists the serotypes for each of the 16 positive urine/genital pairs. Five of eight (63%) of the women with typable urine serotypes had the same typable serotype in the third trimester genital culture, while eight (50%) of the positive genital cultures were in women who had NT urine serotypes. There were two type II serotypes in the urine and one in the genital cultures (Table 1). No urine cultures were positive for type II/c while there were three positive genital cultures. One of the women with type II in her urine had a genital culture positive for type II/c.

**Table 1** Group B streptococcus serotypes from positive first trimester urine and 35–37 weeks genital cultures

| *GBS serotype* | *Positive urine culture, n (%)* | *Positive genital culture, n (%)* |
|---|---|---|
| Ia | 7 (13.2) | 4 (25) |
| Ia/c | 5 (9.4) | 0 |
| Ib | 1 (1.9) | 0 |
| Ib/c | 1 (1.9) | 0 |
| II | 2 (3.8) | 1 (6.3) |
| II/c | 0 | 3 (18.8) |
| III | 17 (32.1) | 5 (31.3) |
| IV/c | 1 (1.9) | 0 |
| V | 3 (5.7) | 2 (12.5) |
| NT | 16 (30.2) | 1 (6.3) |

Total positive urine cultures = 53; total positive genital cultures = 16

**Table 2** Group B streptococcus serotypes of positive first trimester urine and 35–37 week genital culture pairs; $n = 16$

| *Urine culture* | *Genital culture* |
|---|---|
| (2) Ia | (2) Ia |
| Ia | NT |
| II | II/c |
| (2) III | (2) III |
| III | Ia |
| III | II/c |
| NT | Ia |
| NT | II |
| NT | II/c |
| (3) NT | (3) III |
| (2) NT | (2) V |

The other two women with type II/c genital cultures had completely different serotypes (III, NT) in their urine (Table 2).

### DISCUSSION

In this study, only 30.2% of women with asymptomatic first trimester GBS bacteriuria had positive third trimester genital cultures. Of the 30%, 63% had the same typable serotype in the urine and genital cultures. Interestingly, our data provide evidence that some women with asymptomatic first trimester GBS bacteriuria will have a completely different serotype in genital tract cultures at term.

The urine cultures had a relatively large number of NT serotypes. This may have been due to the organism not producing enough antigen to be detected by the assay, the loss of the ability to produce the serotype antigen or the emergence of new serotypes. The small number of typable urine/genital culture pairs is a limitation of the study, but our data suggest the correlation is less than 1:1. Another potential limitation of this study was that the third trimester cultures did not include rectal cultures. The external anal sphincter was cultured but the swabs were not passed through the sphincter into the rectum. If rectal cultures had been obtained, there may have been a greater number of positive third trimester cultures. Indeed our positive predictive value (PPV) of first trimester bacteriuria for a positive culture at 35–37 weeks was only about 50% of the PPV that Edwards and co-workers found for a positive culture at the time of labor[4]. However it should be noted that our culture technique was the same as the one used by Edwards.

The current CDC recommendations for pregnant women with first trimester GBS bacteriuria are based on two studies from 1981[8] and 1985[9]. The first study by Wood and Dillon, had only 14 women with GBS bacteriuria and was an observational study of pregnancy outcome[8]. The second study by Persson and co-workers had only ten women with GBS bacteriuria and evaluated the relationship between significant and asymptomatic GBS bacteriuria[9]. Neither study was designed to detect the rate or degree of genital tract GBS colonization at term.

Our data suggest that first trimester bacteriuria does not automatically equate to heavy genital tract colonization at 35–37 weeks. Possibly 70% of women with first trimester asymptomatic GBS bacteriuria are not at risk for neonatal GBS transmission at term. If all women with first trimester bacteriuria had genital tract cultures at 35–37 weeks, then a large number of women may be able to forgo prophylaxis in labor. For example, assuming 2% of the approximately four million deliveries each year have first trimester GBS bacteriuria, then GBS prophylaxis may not be necessary in 44 560–65 280 women each year. This would help to decrease risks of developing antibiotic resistant GBS, selecting for other penicillin resistant organisms and serious maternal drug reactions. We are not currently advocating the abandonment of the CDC recommendations for intrapartum prophylaxis of women with first trimester bacteriuria; however, our data should provide an impetus for further investigation into whether this is always necessary.

### ACKNOWLEDGEMENT

The authors wish to express their appreciation to John A. Elliott, PhD, of the Centers for Disease Control and Prevention, Atlanta GA, for performing the GBS serotyping.

### REFERENCES

1. Baker CJ. Group B streptococcal infections. *Clinics Perinatol* 1997;24:59–70
2. Anthony BF, Eisenstadt R, Carter J, *et al.* Genital and intestinal carriage of group B streptococci during pregnancy. *J Infect Dis* 1981;143:761–6
3. Schrag S, Gorwitz R, Fultz-Butts K, Schuchat A. Centers for Disease Control and Prevention. Morbidity and mortality weekly report (MMWR): prevention of perinatal group B streptococcal disease. *MMWR Morbid Mortal Wkly Rep* 2002; 51(RR11)
4. Edwards RK, Clark P, Duff P. Intrapartum antibiotic prophylaxis 2: positive predictive value of antenatal group B streptococci cultures and antibiotic susceptibility of clinical isolates. *Obstet Gynecol* 2002;100:540–4
5. Urine culture procedure. In: Isenberg HD, ed. Washington DC: American Society for Microbiology, 1992:1–8
6. Centers for Disease Control and Prevention. Morbidity and mortality weekly report (MMWR): prevention of perinatal group B streptococcal disease: a public health perspective. *MMWR Morbid Mortal Wkly Rep* 1996;45(RR7):1–31
7. Lancefield RC Serologic differentiation of specific types of bovine hemolytic streptococci (group B). *J Exp Med* 1934;59:441–8

8. Wood EG, Dillon HC A prospective study of group B streptococcal bacteriuria in pregnancy. *Am J Obstet Gynecol* 1981;140:515–20
9. Persson K, Christensen KK, Christensen P, Forsgren A, Jorgensen C, Persson PH. Asymptomatic bacteriuria during pregnancy with special reference to group B streptococci. *Scand J Infect Dis* 1985; 17:195–9

RECEIVED 03/31/03; ACCEPTED 08/29/03
